# Changes in IgG oxidoreductase activities in the acute phase and during remission of schizophrenia

**DOI:** 10.1192/j.eurpsy.2026.12200

**Published:** 2026-07-31

**Authors:** T. Amstislavskaya, D. Kazantseva, E. Kornetova, S. Ivanova

**Affiliations:** 1 Scientific Research Institute of Neurosciences and Medicine, Novosibirsk; 2 Laboratory of Molecular Genetics and Biochemistry; 3 Department of Endogenous Disorders, Mental Health Research Institute Tomsk National Research Medical Center Russian Academy of Sciences, Tomsk, Russian Federation

## Abstract

**Introduction:**

Despite decades of research, the pathophysiology of schizophrenia remains incompletely understood. Oxidative stress—resulting from an imbalance between reactive oxygen species and antioxidant defenses—contributes to neuronal damage, neuroinflammation, mitochondrial dysfunction, and impaired dopaminergic and glutamatergic transmission. Antipsychotic treatment may further exacerbate oxidative stress. Catalytic antibodies with superoxide dismutase-like (SOD), catalase-like, and peroxidase-like activity, identified in our laboratory, presumably complement the work of canonical antioxidant systems. Their role in schizophrenia is emerging.

**Objectives:**

To assess SOD-like, catalase-like, and NADPH-dependent peroxidase activities of IgG in schizophrenia patients versus healthy controls, and evaluate their association with disease phase.

**Methods:**

Eighty participants were enrolled: 30 healthy controls and 50 schizophrenia patients (28 acute phase, 22 remission). IgG was purified by affinity chromatography on Protein G‑Sepharose using an ÄKTA pure (GE). Purity was confirmed by Laemmli electrophoresis (4–18% gradient PAGE) and gel filtration (Superdex 200 HR 10/30, pH-shock). Catalase activity was measured at 240 nm (H₂O₂ consumption); SOD activity via inhibition of nitroblue tetrazolium reduction at 560 nm; NADPH-dependent peroxidase activity at 340 nm through NADPH oxidation coupled to glutathione reductase and tert-butyl hydroperoxide. Group comparisons used Mann–Whitney U test (p < 0.05, Statistica 10.0).

**Results:**

SOD-like IgG activity was elevated in remission (p = 0.001). Catalase-like activity IgG was lower in remission versus controls (p = 0.0004) and acute patients (p = 0.002). NADPH-dependent peroxidase activity was increased in both acute (p = 0.0001) and remission (p = 0.017) phases, with highest levels during acute exacerbation.

**Conclusions:**

These results may reflect specific aspects of oxidative stress regulation in schizophrenia. Specifically, we likely observe compensatory activation of IgG peroxidase activity against a background of decreased catalase-like activity. Thus, IgG oxidoreductase activity appears to depend on the course of schizophrenia and may reflect systemic changes in redox processes in patients, although these mechanisms remain poorly understood.

**Disclosure of Interest:**

None Declared

